# Working in the digital economy: A systematic review of the impact of work from home arrangements on personal and organizational performance and productivity

**DOI:** 10.1371/journal.pone.0274728

**Published:** 2022-10-12

**Authors:** Amy Hackney, Marcus Yung, Kumara G. Somasundram, Behdin Nowrouzi-Kia, Jodi Oakman, Amin Yazdani

**Affiliations:** 1 Canadian Institute for Safety, Wellness & Performance, School of Business, Conestoga College Institute of Technology and Advanced Learning, Kitchener, ON, Canada; 2 Department of Occupational Science and Occupational Therapy, University of Toronto, Toronto, ON, Canada; 3 Department of Psychology and Public Health, Centre of Ergonomics and Human Factors, La Trobe University, Bundoora, Australia; 4 School of Public Health and Health Systems, University of Waterloo, Waterloo, ON, Canada; 5 School of Geographic and Earth Sciences, McMaster University, Hamilton, ON, Canada; Shahrood University of Medical Sciences, ISLAMIC REPUBLIC OF IRAN

## Abstract

Work-from-home has become an increasingly adopted practice globally. Given the emergence of the COVID-19 pandemic, such arrangements have risen substantially in a short timeframe. Work-from-home has been associated with several physical and mental health outcomes. This relationship has been supported by previous research; however, these health and safety issues often receive little resources and attention from business perspectives compared to organizational and worker performance and productivity. Therefore, aligning work-from-home practices with business goals may help catalyze awareness from decision makers and serve to effectively implement work-from-home policies. We conducted a review to synthesize current knowledge on the impact of work-from-home arrangements on personal and organizational performance and productivity. Four large databases including Scopus, PubMed, PsychInfo, and Business Source Complete were systematically searched. Through a two-step screening process, we selected and extracted data from 37 relevant articles. Key search terms surrounded two core concepts: work-from-home and productivity/performance. Of the articles published prior to the COVID-19 pandemic, 79% (n = 19) demonstrated that work-from-home increased productivity and performance whereas 21% (n = 5) showed mixed or no effects. Of the articles published during the pandemic, 23% (n = 3) showed positive effects, 38% (n = 5) revealed mixed results, and 38% (n = 5) showed negative effects. Findings suggest that non-mandatory work-from-home arrangements can have positive impacts on productivity and performance. When work-from-home becomes mandatory and full-time, or external factors (i.e., COVID-19 pandemic) are at play, the overall impacts are less positive and can be detrimental to productivity and performance. Results will help foster an understanding of the impact of work-from-home on productivity and performance and inform the development of organizational strategies to create an effective, resilient, and inclusive work-from-home workplace by helping to effectively implement work-from-home policies that are aligned with business goals.

## 1. Introduction

### 1.1 Flexible work arrangements

Since the introduction of the term “telework” in the 1970s [1], the use of flexible working arrangements by organizations has grown in popularity due to improvements in technology [2,3], in support of greater work-life balance [4], and as a means of staying competitive at attracting new generations of workers. Of these flexible work arrangements, work-from-home (WFH), remote work and telework (terms often used interchangeably) have become an increasingly adopted practice across the globe. Since its adoption, researchers have taken a particular interest in understanding the relationship between WFH arrangements and personal and organizational performance, albeit demonstrating mixed results. On one hand, evidence indicates that WFH can have positive impacts including the need for fewer breaks and sick days, greater focus with less distractions [5], increased job autonomy, greater job satisfaction and flexibility to work around life commitments [6]. From an organizational perspective, these factors can have promising results on productivity, employee turnover and cost savings [5–7].

Conversely, some studies have also identified challenges associated with WFH, including blurred lines between work life and home life [8], loss of identity and an inability to unplug [8–11]. When most employees are working from home, organizations may have difficulty building a supportive culture, resulting in reduced motivation and lower job satisfaction [12]. Additionally, WFH can be complicated by reduced access to resources (e.g., technical assistance, equipment) and opportunities for social interaction [6,13,14]. Such negative impacts have been associated with adverse individual outcomes such as anxiety, problems with task completion and irritability [15], as well as decreased productivity, reduced motivation, and increased stress [6,16–19]. From an organizational perspective, WFH may reduce productivity, increase training costs, and decrease opportunities for knowledge sharing, mentoring, and networking [20–22].

Despite the rise in WFH arrangements, the scientific literature reveals mixed evidence on its effectiveness, and it is clear the relationship between WFH and personal and organizational productivity and performance is complex. Recent work has highlighted the need for formalized organizational policies to protect employees and ensure positive and productive experiences for both the worker and the organization, whilst acknowledging the need for future research [23]. To the best of our knowledge, no study has been undertaken which comprehensively reviews the literature on the impacts of WFH arrangements on personal and organizational performance and productivity. Therefore, the objective of this project was to synthesize current knowledge on the impacts of WFH for both personal and organizational productivity and performance.

### 1.2 WFH and the COVID-19 pandemic

This review is extremely timely given the declaration of COVID-19 as a global pandemic on March 11, 2020 by the World Health Organization (WHO), causing millions of people and organizations around the world to have a sudden and radical change in the way they work. In Canada, we have observed a drastic jump from 4% of the working population working from home in 2016 to an estimated 32% as of 2021 [24]. Similar trends have been observed around the globe. In the United States, 17% of the workforce worked from home prior to the pandemic; however, the proportion increased to nearly 50% during COVID-19 [25,26]. Europe saw nearly 40% of its workers in a WFH arrangement as compared to 10% previously [27], and Australia reports a jump from under 20% to nearly 50% [28].

Unfortunately, many organizations may not have been prepared for the sudden and drastic shift to remote working, with insufficient or non-existent policies and recommendations in place to support the transition to WFH. Many organizational policies supporting employees in WFH arrangements are considered organizational health and safety matters with restricted influence and resources [29,30], making them difficult to implement [31]. However, aligning WFH policies with organizational goals, such as productivity and performance, can help position these health and safety issues at the attention of decision-makers and provide more resources and attention within an organization [32,33].

Prior to the COVID-19 pandemic, many WFH arrangements were voluntary and available on a part time basis (i.e., workers were able to WFH for a subset of their working hours per week), which allowed individuals to choose solutions that worked best for their needs. Most literature on the impacts of WFH on productivity and performance reflect voluntary arrangements rather than mandatory working at home. Given the sudden necessity of organizations and their employees to shift to mandatory and full-time remote work, in an effort to reduce the spread of COVID-19, an urgent need exists to understand the complex relationship between WFH arrangements and personal and organizational productivity and performance.

A recent rapid review identified several negative health outcomes influenced by the degree of organizational and peer/colleague support, social connectedness, and levels of work-family conflict due to WFH arrangements [23]. A gender-focused analysis also revealed that women were less likely to experience improved health outcomes from WFH arrangements [23]. The findings supported the need for formalized organizational policies but noted many gaps in the available literature, in particular the influence of mandatory WFH arrangements on employees’ health and well being. By undertaking the current review, we have helped establish an understanding about the relationship between WFH and personal and organizational productivity and performance through the context of both WFH arrangements prior to, and during the COVID-19 pandemic. This work will ensure workers and their organizations are equipped with the knowledge, resources, and recommendations to maintain a productive and healthy workforce.

## 2. Methods and analysis

We conducted a systematic review to synthesize current knowledge on the effects of WFH arrangements on personal and organizational productivity and performance. Systematic reviews are performed with the goal of delivering a clear and comprehensive overview of evidence and identify research gaps in the field [34].

### 2.1 Search

Our search included four large databases: Scopus, PubMed, PsychInfo, and Business Source Complete. We identified two core concepts that encompassed the key aspects of our research question, those relating to work from home (e.g., “telecommute”) and work-related outcomes (e.g., “performance”, “productivity”). We worked with a librarian to generate a list of relevant search terms for each concept (Table 1). The Boolean operator “OR” was used between search terms within each concept, and the Boolean operator “AND” was used across concepts.

**Table 1:** Search terms.

**Work from home terms**	work from home, work at home, telecommute, virtual work, remote work, distributed work, telework
**Productivity and performance terms**	work performance, job satisfaction, efficiency, productivity, job satisfaction, work satisfaction, organizational objectives, presenteeism, absenteeism, innovation, cost saving, turnover, work life balance, sales, quality, competitive, task completion, collaboration, work culture, employee morale, customer relations, customer satisfaction
**Exclusions**	homework, schoolwork, teleoperation, telemental, telemetry, telemedicine, homecare, residential facilities, domestic work, residential care, aged care, elder care, childcare
**Limitations**	Must be written in English language, published 2010 and later, and in peer reviewed journals

To be included in the review, studies were required to focus on adult employees working from home in some capacity, and to evaluate the work arrangement on personal or organizational productivity or performance. To ensure we captured the overarching question, we did not limit the search to a particular industry, sector, or region. However, articles were required to be written in English, published between January 2010 and February 2021, and published in peer-reviewed journals. January 2010 was chosen to coincide with the US Telework Enhancement Act of 2010. Signing of the act was thought to have encouraged strategic intervention for supporting organizational effectiveness, and as such, scientific evaluation of this effectiveness followed.

### 2.2 Study selection

The methodological approach followed Arksey and O’Malley [35] and Yung et al [36]. Our review involved a two-step screening process: (1) title and abstract screening, and (2) full text review and data extraction. All titles and abstracts identified from our search strategy were retrieved and uploaded into Rayyan QCRI, a web and mobile app that compiles and organizes articles for systematic reviews [37]. Duplicate articles were removed from further screening using a feature in the Rayyan program, which automatically removes duplicate articles. For the first screening step, a screening tool of inclusion/exclusion criteria was developed. We excluded articles with a primary focus of work-life balance, job satisfaction, or organizational commitment without context to productivity or performance. We also excluded articles where the WFH population were full time students and not organizational employees. To reduce the risk of selection bias, two reviewers independently screened the first 5% of the titles and abstracts to confirm inter-rater agreement. Any discrepancies in decisions were discussed until consensus was reached. After discussion, no difference of opinions remained. Each reviewer then independently screened the titles and abstracts of the remaining articles.

### 2.3 Data collection

The remaining articles were subjected to our second screen, full text review and data extraction. Using a standardized form in Microsoft Excel, we gathered study information (i.e., title, authors, publication year, journal, study type, country/region, sample size, COVID-19 specific research, and industry of focus) and detailed study methodology and findings including target measures (i.e., personal, organizational, or both), outcomes (i.e., productivity and performance), associated direct outcomes (i.e., turnover, cost savings, work intensification, distractions, and absenteeism/sick days), and associated indirect outcomes (i.e., job satisfaction, work-life balance, work engagement, organizational commitment, stress, and motivation) (Table 2). We broadly categorized articles as 1) organizationally focused, 2) personally focused or 3) both, determined by evaluating the objective of the study, the variables measured and the application of the findings. Table 2 outlines the screening extraction process for these full-text reviews. The first five articles were screened by three reviewers to ensure accuracy of data extraction. Discrepancies were discussed until consensus was reached. The reviewers then independently extracted data from the remaining articles.

**Table 2:** Details of the second screen extraction process from full-text reviews.

Extraction Type	Category	Definitions
Study information	Title, authors, publication year, journal, study type, country/region, sample size, COVID-19 specific research, industry of focus.	
Target measures	Personal	Measures of productivity or performance focused on the employee’s job, role, task, or responsibility.
	Organizational	Measures of productivity or performance focused on the overall output or quality of goods and services impacting the organization.
	Both	Measures of productivity and performance focused on both personal and organizational level impacts.
Outcomes	Performance	Assesses the ability and quality of a completed job, role, task, or responsibility.
	Productivity	Efficiency of production of goods or services expressed by some measure.
Associated direct outcomes	Turnover	Rate at which employees leave a workplace.
	Cost savings	Set of actions or policies that reduce the historical or expected cost of a given transaction.
	Work intensification	Increasing amount of effort an employee must invest from increased economic pressure and societal changes.
	Distractions	Process of diverting someone’s attention away from his/her desired area of focus.
	Absenteeism/sick days	Employee absence from work for lengths beyond what is considered an acceptable time span (absenteeism); paid time off that workers can use to stay home to address health needs (sick days).
Associated indirect outcomes	Job satisfaction	Worker’s contentedness with their job. Can be measured in cognitive, affective, or behavioural components.
	Work-life balance	Equilibrium between personal life and career work. How people manage time spent at and outside of work.
	Work engagement	The harnessing of organization member’s selves to their work roles: in engagement, people employ and express themselves physically, cognitively, emotionally, and mentally during role performances.
	Organizational commitment	An employee’s attachment to the organization. Includes affective commitment, continuance commitment and normative commitment.
	Stress	State of mental or emotional strain or tension resulting from adverse or demanding circumstances.
	Motivation	Set of internal and/or environmental forces that originate within individuals, and in their environment, to initiate work-related behaviours.

After our second screening process was complete, we returned to the data for a further screening step surrounding the type of measures used in the articles. As part of the initial search, to ensure we were as inclusive as possible in addressing the productivity and performance measures, we had extracted data from articles that measured variables associated with productivity and performance in addition to these specific terms. This list of summary measures included variables that both directly related to productivity and performance (i.e., turnover, cost savings), and indirectly related the measures (i.e., job satisfaction, organizational commitment). This allowed us to further screen the articles to ensure that articles that did not contain any of the direct measures, including productivity and performance were excluded. In Table 2, we outline the specific details that were extracted from each article.

### 2.4 Quality appraisal

The studies were appraised by two reviewers independently using the JBI Critical Appraisal Tools [38]. JBI Checklist for Quasi-Experimental Studies (JBI-QE) included nine questions and was used to appraise quasi-experimental studies. Cross-sectional studies were appraised using the JBI Checklist for Analytical Cross-Sectional Studies (JBI-ACS), which comprised eight questions. Both tools consisted of questions regarding study methodology including statistical analyses, confounding variables, participants, and outcome reliability. Pre-determined cut-off score of 5 was established for the appraisal [39]. Discrepancies in the quality appraisal scores were discussed until consensus was reached.

## 3. Results

### 3.1 Overall results of all relevant articles

**Fig 1:**
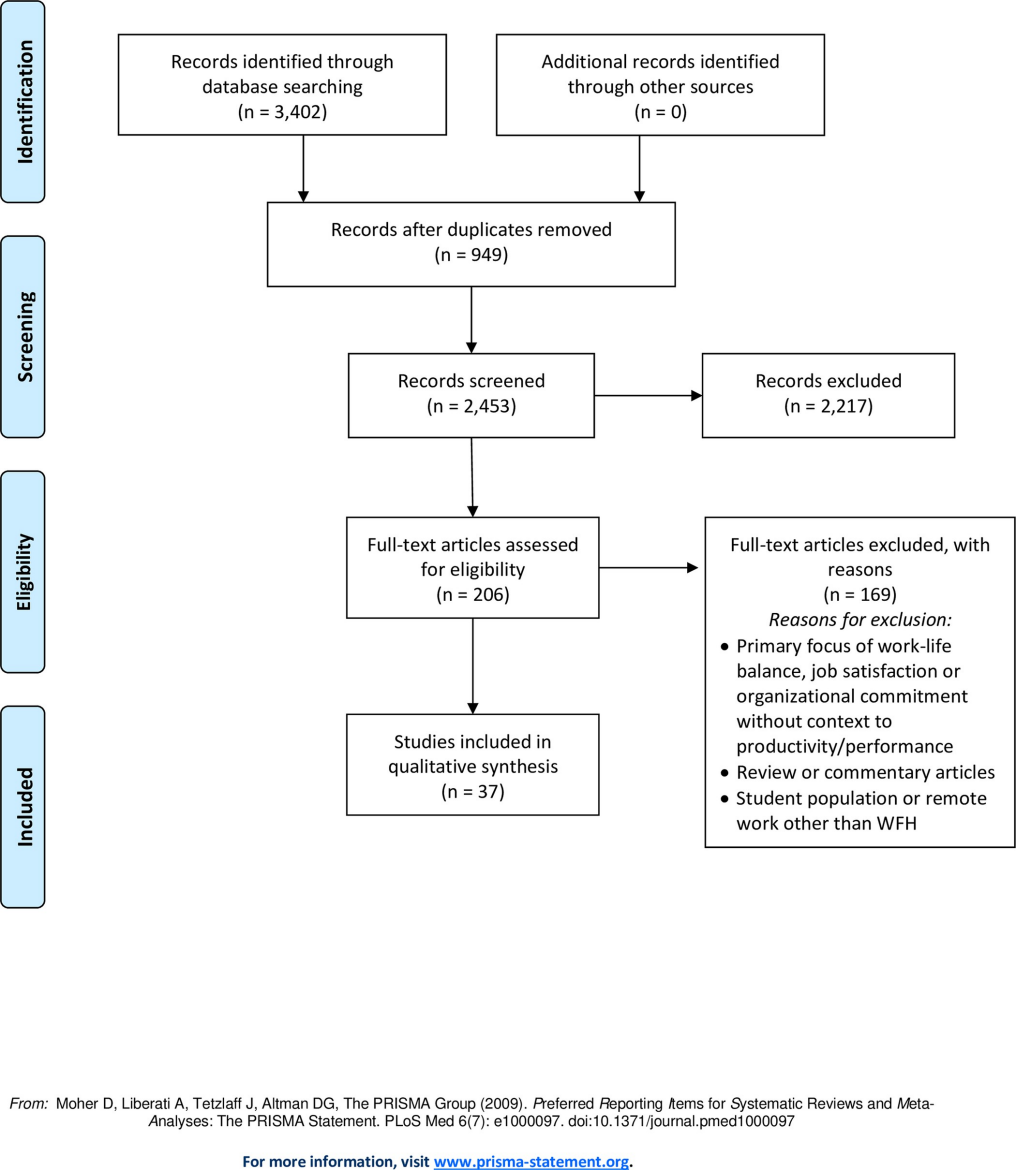
Flow diagram of identifying, screening, and extracting data from obtained articles using PRISMA (data in S1 Checklist).

We retrieved a total of 3,402 articles and removed 949 duplicates, which left 2,453 articles for title and abstract screening. Of the 206 studies retained for full text screening and data extraction, 37 were included (Fig 1).

The retained articles are summarized by study characteristics in Table 3. Many of the articles used a survey-based design (62%), followed by interviews (22%), and experimental designs (16%). Although a variety of countries were investigated, the United States of America (n = 10) was disproportionally represented compared to all other countries, which included one to four articles each. Articles also covered a range of industries, with many articles including participants from more than one industry (n = 16).

**Table 3:** Summary of articles by study characteristics.

Study Design	Authors, Year	Sample Size (% female)	COVID-19?	Country	Industry	Measures	Quality Appraisal Score
Survey	Aboalmaali, Abedi & Ketabi, 2015 [40]	316 (53%)	N	Iran	Government	Performance, cost saving, distractions, job satisfaction, organizational commitment, motivation	JBI-ACS 6
	Aboelmaged & Subbaugh, 2012 [41]	199 (32%)	N	Egypt	Variety	Productivity, job satisfaction, organizational commitment	JBI-ACS 6
	Atiku, Jeremiah & Boateng, 2020 [42]	473 (undefined)	Y	Africa	Service	Productivity, WLB, stress	JBI-ACS 6
	Baard & Thomas, 2010 [43]	63 (46%)	N	South Africa	Telecommunications Finance	Productivity, cost saving, work intensification, distractions, absenteeism/sick days, job satisfaction, WLB, organizational commitment, morale	JBI-ACS 6
	Bolisani, Scarso, Ipsen, Kirchner, Hansen, 2020 [44]	1,000 (40%)	Y	Italy	Variety	Productivity, work intensification	JBI-ACS 6
	Chapman & Thamrin, 2020 [45]	163 (72%)	Y	Australia	Academia	Productivity	JBI-ACS 7
	Dixit, Chinnam, & Singh, 2020 [46]	300 (undefined)	Y	USA	Defense	Productivity, work intensification, job satisfaction	JBI-ACS 6
	Drumea, 2020 [47]	N/A	Y	Unknown	Variety	Productivity, WLB, stress	JBI-ACS 6
	Feng & Savani, 2020 [48]	286 (49%)	Y	USA	Variety	Productivity, job satisfaction	JBI-ACS 8
	Gajendran, Harrison, & Delaney-Klinger, 2014 [49]	323 (53%)	N	USA	Variety	Performance	JBI-ACS 8
	Golden, & Gajendran, 2019 [50]	273 (undefined)	N	USA	Unknown	Performance	JBI-ACS 7
	Greer, & Payne, 2014 [51]	342 (48%)	N	USA	Accounting	Performance, WLB, organizational commitment	JBI-ACS 6
	Kazekami, 2019 [52]	9,200 (31%)	N	Japan	Variety	Productivity, work intensification, job satisfaction, WLB, stress	JBI-ACS 6
	Mirela, 2020 [53]	57 (undefined)	Y	Romania	Variety	Productivity, distractions, job satisfaction	JBI-ACS 6
	Ralph et al., 2020 [54]	225 (18%)	Y	Global	IT/Software	Productivity	JBI-ACS 7
	Tanpipat, Wen Lim & Deng, 2021 [55]	414 (59%)	Y	Thailand	Variety	Productivity, work intensification, organizational commitment	JBI-ACS 6
	Tavares, Santos, Diogo & Ratten, 2020 [56]	359 (59%)	Y	Portugal	Variety	Productivity, work intensification, WLB	JBI-ACS 6
	Torten, Reaiche, & Caraballo, 2016 [57]	400 (49%)	N	USA	Variety	Productivity, performance, job satisfaction	JBI-ACS 6
	Toscano, & Zappala, 2020 [58]	265 (63%)	Y	Italy	Variety	Productivity, work-life balance, stress	JBI-ACS 6
	Turetken, Jain, Quesenberry, & Ngwenyama, 2011 [59]	89 (51%)	N	USA, Canada	Variety	Productivity, performance, job satisfaction	JBI-ACS 6
	Tustin, 2014 [60]	310 (undefined)	N	South Africa	Academia	Productivity, cost saving, distractions, absenteeism/sick days, job satisfaction, WLB, stress, morale	JBI-ACS 6
	Vega, Anderson, & Kaplan, 2015 [61]	180 (59%)	N	USA	Government	Performance, job satisfaction	JBI-ACS 6
	Virick, DaSiliva & Arrington, 2010 [62]	88 (25%)	N	USA	Telecommunications	Performance, job satisfaction	JBI-ACS 6
Interview	Coenen & Kok, 2014 [63]	7 (undefined)	N	Unknown	Telecommunications	Performance	JBI-ACS 6
	Davidescu, Apostu, Paul, & Casuneanu, 2020 [64]	220 (45%)	N	Romania	Variety	Productivity, performance, job satisfaction, motivation	JBI-ACS 6
	Grant, Wallace, & Spurgeon, 2013 [65]	11 (64%)	N	United Kingdom	Variety	Productivity, distractions, WLB, motivation	JBI-ACS 6
	Karia & Asaari, 2016 [66]	N/A	N	Malaysia	Skilled Trades or Construction	Productivity	JBI-ACS 6
	Neirotti, Paolucci, & Raguseo, 2012 [67]	1,134 (undefined)	N	Italy	Variety	Productivity	JBI-ACS 6
	Tietze & Nadin, 2011 [68]	7 (100%)	N	Unknown	Tax	Productivity, organizational commitment	JBI-QE 6
	Viorel, Ionut, & Andreea-Oana, 2018 [69]	220 (45%)	N	Romania	Academia Service	Productivity, performance, job satisfaction, motivation	JBI-ACS 8
	Wang, Liu, Qian & Parker, 2020 [70]	661 (52%)	Y	China	Variety	Productivity, performance	JBI-ACS 6
Experimental	Bloom, Liang, Roberts & Ying, 2013 [5]	249 (undefined)	N	China	Call center	Productivity, performance, turnover, cost saving	JBI-QE 7
	Delanoeije & Verbruggen, 2020 [71]	78 (undefined)	N	Belgium	Skilled Trades or Construction	Performance, WLB, work engagement, stress	JBI-QE 7
	Dutcher, 2012 [72]	125 (48%)	N	USA	Academia	Productivity	JBI-QE 6
	Hardy, Marcolino, & Fontanari, 2021 [73]	100 (undefined)	Y	unknown	Unknown	Productivity	JBI-QE 6
	Nijp, Beckers, van de Voorde, Geurts, & Kompier, 2016 [74]	2,912 (36%)	N	Netherlands	Finance Insurance	Performance, work intensification, job satisfaction, WLB, organizational commitment	JBI-QE 8
	Sherman, 2018 [75]	187 (100%)	N	United Kingdom	Life Sciences	Performance, work intensification, absenteeism, job satisfaction, WLB	JBI-QE 6

Note: (WLB = work-life balance).

Specific industries of interest included academia, telecommunications, skilled trades, service, government, finance, insurance, IT/software, life sciences, defence, call centers, tax, and accounting. Sample sizes varied based on the study design: survey-based articles ranged from 57 to 9,200 participants, interview-based studies ranged from 7 to 1,134 participants, and experimental studies included 78 to 2,912 subjects. Twenty-five articles provided a gender/sex split of their sample size, with the percentage of female participants ranging from 18%-100% of the sample (average 51.9%). Twelve articles either did not provide a gender/sex breakdown or it was not applicable because the experiment did not include individual participants.

We also categorized the articles based on whether the research was conducted during the COVID-19 pandemic. Articles were categorized as either COVID-19 specific (examined during the COVID-19 pandemic) or “pre-pandemic” articles (i.e., examined prior to March 11, 2020). This categorization separated articles where WFH was a mandatory, full-time arrangement as required by government lockdowns and where unique factors associated with the ongoing COVID-19 pandemic may influence the results. In total, 65% of the articles were conducted during “pre-pandemic” times (n = 24), and 35% were conducted during the COVID-19 pandemic (n = 13).

For inclusion in this study, all articles were required to have specifically measured either productivity or performance, at either the personal or organizational level (or both). As previously described, several additional measures were identified as relevant to establishing an understanding of the effects of WFH on productivity and performance.

**Fig 2:**
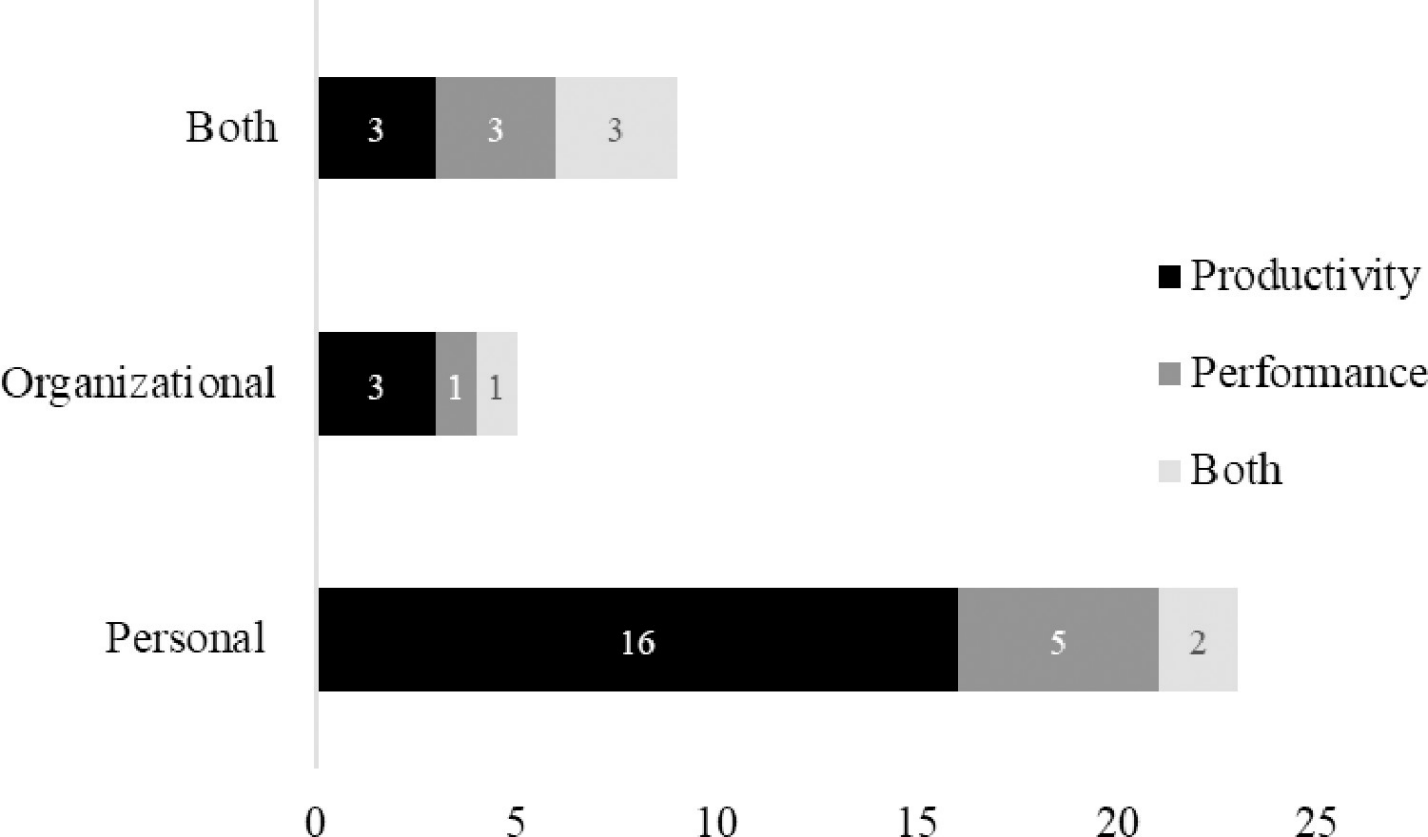
Number of articles by target measure and outcome measure.

Table 3 also lists the specific productivity and performance measures examined in each article. Overall, 62% (n = 23) of the articles focused on personal target measures: productivity outcomes (n = 15), performance outcomes (n = 6), or both outcomes (n = 2). Meanwhile, only 14% (n = 5) articles were concerned with organizational target measures: productivity (n = 3), performance (n = 1), both outcomes (n = 1); and 24% (n = 9) focused on both personal and organizational target measures: productivity (n = 3), performance (n = 3), and both (n = 3). Fig 2 displays the number of articles by target measure (i.e., productivity, performance or both) as they relate to the outcome measures (i.e., personal, organizational or both).

Table 4 provides a summary of the number of articles examining specific measures. In total, productivity measures were examined in 27 of the 37 articles and performance was measured in 16 of the 37 articles. Direct measures included: turnover (n = 1), cost savings (n = 4), work intensification (n = 8), distractions (n = 6), and absenteeism/sick days (n = 3). We also captured the indirect productivity and performance measures examined in the studies. These included job satisfaction (n = 17), work-life balance (n = 13), work engagement (n = 1), organizational commitment (n = 7), stress (n = 6), motivation (n = 4), and employee morale (n = 2).

**Table 4:** Number of articles examining each type of measure, and example questions involved.

Measure	# of Articles	Type of Questions
Performance	16	Self-reported: overall performance during daily performance Self-reported: overall job or extra role performance Self-reported: perceived quality of work Evaluation: supervisor performance rating of employees Quantitative: number of tasks completed Open-ended interview questions
Productivity	27	Self-reported: overall productivity on tasks Self-reported: comparative productivity pre and post Quantitative: number of tasks completed Quantitative: ratio (annual income/working hours per week) Open-ended interview questions
Turnover	1	Quantitative: rate at which employees leave a workplace
Cost Savings	4	Self-reported: perceived changes in monthly expenses Quantitative: value attributed per employee based on performance measures and turnover rate
Work intensification	7	Self-reported: perceived increase in working hours Self-reported: perceived changes in work demand Quantitative: number of hours worked during week, evenings, or weekends Open-ended interview questions
Distractions	6	Self-reported: number of distractions during working hours
Absenteeism / sick days	3	Self-reported: changes in the number of sick days taken
Job satisfaction	17	Self-reported: overall satisfaction with work Self-reported: willingness to recommend to others Self-reported: comparative job satisfaction pre and post
Work-life balance	13	Self-reported: perceived changes in overall work-life balance or and conflicts between work and home Self-reported: prevalence of time-based/strain-based conflicts Semi-structured and open-ended interview questions
Work engagement	1	Self-reported: overall engagement and excitement about work
Organizational commitment	7	Self-reported: affective commitment/loyalty to company Self-reported: turnover intention Open-ended interview questions
Stress	6	Self-reported: general, daily, or work-related levels of stress
Motivation	4	Self-reported: motivation towards completing work tasks Open-ended interview questions
Morale	2	Self-reported: overall employee morale

Retained articles varied by the type of questions used for each measure. Twenty studies relied on self-reported or perceived outcomes of specific measures such as perceived performance while in a WFH arrangement, or self-reported improvements in productivity. Several studies asked participants to rate their level of agreement with specific statements for measures such as job satisfaction or work-life balance. Less commonly, articles included quantifiable evaluations of specific measures, such as the number of tasks completed in a certain length of time or the total cost savings per employee. Examples of the types of questions included for each measure are described in Table 4.

Quality appraisal scores are presented in Table 3. JBI Checklist for Quasi-Experimental Studies was used to appraise seven articles whereas the remaining 30 articles were appraised using the JBI Checklist for Analytical Cross-Sectional Studies. The median appraisal score was 6. According to the JBI Checklist for Quasi-Experimental Studies, 11% of the articles (n = 4) scored 6, 5% (n = 2) scored 7, and 3% (n = 1) scored 8. Based on the JBI Checklist for Analytical Cross-Sectional Studies, 65% of the articles (n = 24) scored 6, 8% (n = 3) scored 7, and the remaining 8% (n = 3) scored 8.

### 3.2 Results from pre-pandemic specific articles

**Fig 3:**
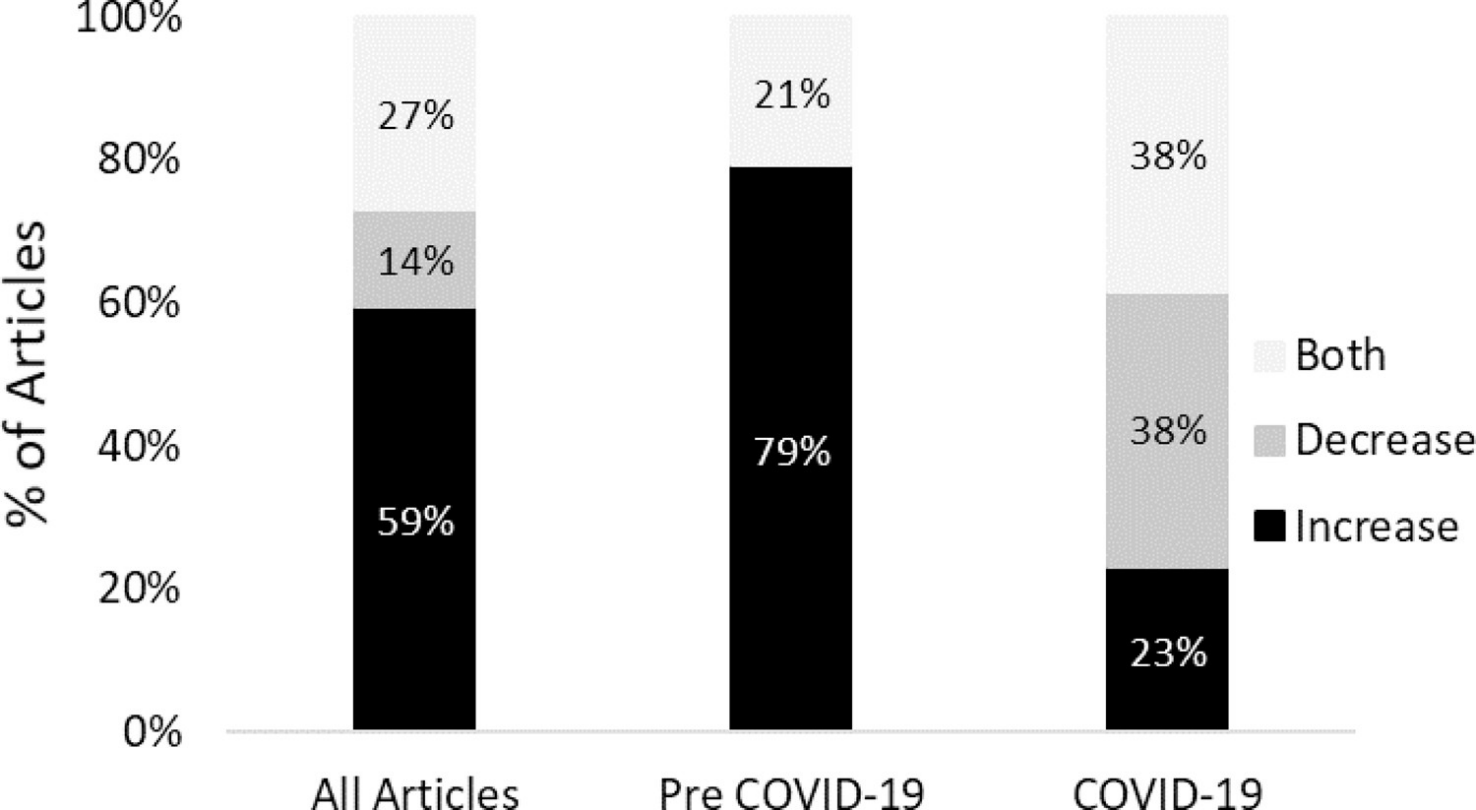
Percentage of articles by effect on performance/productivity.

We summarized results based on the effect of WFH arrangements. Table 5 displays these outcomes for “pre-pandemic articles”. In general, these articles found a positive effect of WFH on productivity and performance. Of the pre-pandemic specific articles, 79% (n = 19), reported that WFH increased or improved personal or organizational productivity and performance, whereas 21% (n = 5) demonstrated both an increase and decrease or no effect (Fig 3). No “pre-pandemic” articles reported negative impacts on productivity or performance. Several associated measures were positively affected in all studies examining them: reduced turnover rates (n = 1) and stress (n = 4), increased cost savings (n = 4), work engagement (n = 1) and morale (n = 2). Further, the majority also demonstrated positive impacts on the following: increased job satisfaction (n = 10), better work-life balance (n = 7), reduced absenteeism (n = 3), greater organizational commitment (n = 4), and increased motivation (n = 3). Interestingly, despite improvements in productivity and performance, articles demonstrated mixed results on whether WFH increased or decreased the number of distractions (n = 4), and work intensification saw both effects (n = 2).

**Table 5:** Outcomes of “pre-pandemic” articles (N = 24).

Article	Productivity	Performance	Turnover	Cost Saving	Work Intensification	Distractions	Absenteeism	Job Satisfaction	Work-Life Balance	Engagement	Organizational Commitment	Stress	Motivation	Morale
Baard & Thomas, 2010 [43]	↑			↑	↑	↓	↓	↑	↑		↑	↓		↑
Virick, DaSiliva & Arrington, 2010 [62]		↑						↑						
Tietze & Nadin, 2011 [68]	↑								↑		↓			
Turetken, Jain, Quesenberry & Ngwenyama, 2011 [59]—task interdependence & tenure effect	↕	↕						↑						
Aboelmaged & Subbaugh, 2012 [41]	↑							*			*			
Neirotti, Paolucci, & Raguseo, 2012 [67]	↑													
Dutcher, 2012 [72]	↕													
Bloom, Liang, Roberts & Ying, 2013 [5]	↑	↑	↓	↑				↑						
Grant, Wallace, & Spurgeon, 2013 [65]	↑					↓			↑				↕	
Aboalmaali, Abedi & Ketabi, 2015 [40]		↑		*		*		*			*		*	
Coenen & Kok, 2014 [63]		↑												
Gajendran, Harrison, & Delaney-Klinger, 2014 [49]		↑												
Greer, & Payne, 2014 [51]		↑							↑		↑			
Tustin, 2014 [60]	↑			↑		↓	↓	↑	↑			↓		↑
Vega, Anderson, & Kaplan, 2015 [61]		↑						↑						
Karia & Asaari, 2016 [66]	↑													
Nijp, Beckers, van de Voorde, Geurts & Kompier, 2016 [74]		Ø			↑			Ø	Ø		Ø			
Torten, Reaiche, & Caraballo, 2016 [57] -experience, tenure, days teleworked/week	↕	↕						↕						
Sherman, 2018 [75] -caregiver and gender effects		↑			Ø		Ø	↕	↕					
Viorel, Ionut, & Andreea-Oana, 2018 [69]	↑	↑						↑					↑	
Golden, & Gajendran, 2019 [50]		↑												
Kazekami, 2019 [52]	↕				*			↑	↑			↓		
Davidescu, Apostu, Paul, & Casuneanu, 2020 [64]	↑	↑						↑					↑	
Delanoeije & Verbruggen, 2020 [71]—daily effects		↑							↑	↑		↓		

Note: (↑ increased, ↓ decreased, * moderating effect, ↕ increase/decrease, Ø no effect). For articles reflecting group effects (indicated in the author column), outcome symbols represent all effects observed.

Of the five articles reporting both positive and negative or no effects of WFH on productivity and performance, work intensification increased (n = 2), stress decreased (n = 1), and organizational commitment was not affected (n = 1). Mixed results (both positive and negative) were identified for the impact of WFH on job satisfaction (n = 4), and work-life balance (n = 2).

A description of the specific main outcomes for each study can be found in Table 6.

**Table 6:** Summary of “pre-pandemic” articles including effect on productivity/performance, author/year and the main findings (N = 24).

Effect on Productivity / Performance	Author	Main Finding
Increase	Baard & Thomas, 2010 [43]	Increased productivity, job satisfaction, morale, organizational commitment, work-life balance. Decreased distractions, stress, and sick days. Longer working hours and higher cost savings.
	Virick, DaSiliva & Arrington, 2010 [62]	Increased performance positively associated with increased job satisfaction
	Tietze & Nadin, 2011 [68]	Increased productivity and work-life balance but decreased organizational commitment.
	Aboelmaged & Subbaugh, 2012 [41]	Job satisfaction and organizational commitment increased perceived productivity.
	Neirotti, Paolucci, & Raguseo, 2012 [67]	Increased productivity.
	Bloom, Liang, Roberts & Ying, 2013 [5]	Increased performance (13%), productivity, job satisfaction. Decreased turnover (50% drop) and more cost savings ($2,000/employee).
	Grant, Wallace, & Spurgeon, 2013 [65]	Increased productivity, and work-life balance. Motivation increased for some participants but decreased for others.
	Aboalmaali, Abedi & Ketabi, 2015 [40]	Increased performance. Factors with a positive relationship with performance include organizational commitment, cost saving, motivation, job satisfaction and focus.
	Coenen & Kok, 2014 [63]	Increased performance and improved product quality.
	Gajendran, Harrison, & Delaney-Klinger, 2014 [49]	Increased performance (task and contextual). strong supervisor-employee relationship increased individual effectiveness
	Greer, & Payne, 2014 [51]	Increased performance and work-life balance. Decreased turnover intention (organizational commitment) with specific strategies to overcome challenges.
	Tustin, 2014 [60]	Increased productivity, job satisfaction, work-life balance, and morale. Decreased travel cost (more cost saving), distractions, stress, and absenteeism.
	Vega, Anderson, & Kaplan, 2015 [61]	Increased performance, increased job satisfaction
	Karia & Asaari, 2016 [66]	Increased productivity and competitive advantage with proper technology adoption.
	Sherman, 2018 [75]	Increased performance increased overall, but most beneficial to mothers. Increased job satisfaction for men and was unchanged in women. Decreased work-family conflict for mothers but not fathers or nonparents. No change in sick leave or work intensification.
	Viorel, Ionut, & Andreea-Oana, 2018 [69]	Increased performance and productivity, job satisfaction and motivation
	Golden, & Gajendran, 2019 [50]	Increased performance. More extensive telecommuting is related to greater performance.
	Davidescu, Apostu, Paul, & Casuneanu, 2020 [64]	Increased performance, organizational performance, job satisfaction, and motivation.
	Delanoeije & Verbruggen, 2020 [71]	Increased performance and engagement on days of teleworking compared to office work, but only on days when employee was teleworking. Decreased stress.
Both or no change	Turetken, Jain, Quesenberry, & Ngwenyama, 2011 [59]	Experience teleworking, communication skills and task interdependence determines success (productivity and performance). Tenure only positively correlates with job satisfaction.
	Dutcher, 2012 [72]	Increased productivity with creative tasks but decreased with dull tasks.
	Nijp, Beckers, van de Voorde, Geurts, & Kompier, 2016 [74]	No change in performance, organizational commitment, job satisfaction, or work-life balance, but work intensification increased.
	Torten, Reaiche, & Caraballo, 2016 [57]	Experience teleworking and tenure positively relate to productivity but not performance or job satisfaction. Number of days teleworking per week impacts performance and satisfaction but not productivity.
	Kazekami, 2019 [52]	Increased productivity with appropriate working hours but decreases with too many hours (work intensification). Increased life satisfaction (work-life balance) and job satisfaction Decreased stress. These three factors do not impact productivity.

### 3.3 Results from COVID-19 specific articles

We completed a similar process for the “COVID-19 specific” articles and summarized the outcomes in Tables 7 and 8. Contrary to the general positive impact of WFH on productivity and performance in majority of the “pre-pandemic” articles, majority of the “COVID-19” literature showed both positive and negative (mixed) results (see Table 7). In COVID 19 literature, only 23% of articles reported positive impacts of WFH on productivity and performance, whereas 38% demonstrated mixed results and another 38% reported negative impacts. For example, Atiku and colleagues [42] revealed greater productivity and work-life satisfaction (positive) whereas Toscano and Zappala [58] observed lower productivity and job satisfaction as well as greater stress in WFH employees (negative). On the other hand, Feng and Savani [48] showed that productivity and job satisfaction increased for men but decreased for women (mixed). Only one “COVID-19” article focused on both performance and productivity, reporting an overall decrease in both measures, while the remaining 12 articles only focused on productivity.

**Table 7:** Outcomes of COVID-19 articles (N = 13).

Article	Productivity	Performance	Turnover	Cost Saving	Work Intensification	Distractions	Absenteeism	Job Satisfaction	Work-Life Balance	Engagement	Organizational Commitment	Stress	Motivation	Morale
Atiku, Jeremiah & Boateng, 2020 [42]	↑								↑					
Bolisani, Scarso, Ipsen, Kirchner, Hansen, 2020 [44]	↕				↓							↑		
Mirela, 2020 [53]	↓					↕		↓						
Chapman & Thamrin, 2020 [45]	↑													
Dixit, Chinnam, & Singh, 2020 [46]	↕				↕			↑						
Drumea, 2020 [47]	↕								*			*		
Feng & Savani, 2020 [48]—gender effects	↕							↕						
Ralph et al., 2020 [54]	↓													
Tavares, Santos, Diogo & Ratten, 2020 [56]	↓				↑				↑					
Toscano, & Zappala, 2020 [58]	↓							*				*		
Wang, Liu, Qian & Parker, 2020 [70], effects of social support	↓	↓				↓			↓					
Hardy, Marcolino, & Fontanari, 2021 [73]	↕													
Tanpipat, Wen Lim & Deng, 2021 [55]—effect of organizational norm	↑				Ø						↑			

Note: (↑ increased, ↓ decreased, * moderating effect, ↕ both increase/decrease, Ø no effect).

**Table 8:** Summary of COVID-19 articles including effect on productivity/performance, author/year and the main findings (N = 13).

Effect on Productivity / Performance	Article	Main Finding
Increase	Atiku, Jeremiah & Boateng, 2020 [42]	Increased productivity and work-life satisfaction (work-life balance).
	Chapman & Thamrin, 2020 [45]	Increased productivity. Job experience results in greater productivity.
	Tanpipat, Wen Lim & Deng, 2021 [55]	Organizational norm increased productivity, and organizational commitment. Work demands (work intensification) is unaffected.
Both or no change	Bolisani, Scarso, Ipsen, Kirchner, Hansen, 2020 [44]	Productivity increased with online meetings but decreased because of continuous online connection. Increased stress. Less demanding (work intensification).
	Dixit, Chinnam, & Singh, 2020 [46]	Decreased productivity and increased work intensification initially and then increased productivity and decreased work intensification after adapting. Increase job satisfaction.
	Drumea, 2020 [47]	Productivity was negatively mediated by increased anxiety, isolation, confinement (categorized as stress), but positively mediated by improved work-life balance.
	Feng & Savani, 2020 [48]	Decreased productivity and job satisfaction for women. Increased productivity and job satisfaction for men.
	Hardy, Marcolino, & Fontanari, 2021 [73]	Introverts and extroverts’ productivity are affected differently by social isolation and social distancing resulting from the COVID-19 pandemic.
Decrease	Mirela, 2020 [53]	More people were very dissatisfied than were very satisfied with their job; 57% of people said WFH negatively influences productivity; 43% do not keep regular hours; 50% report more distractions.
	Ralph et al., 2020 [54]	Decreased productivity.
	Tavares, Santos, Diogo & Ratten, 2020 [56]	Increased work intensification and work-life balance. Decreased productivity.
	Toscano, & Zappala, 2020 [58]	Decreased job satisfaction and productivity (result of social isolation). Increased stress.
	Wang, Liu, Qian & Parker, 2020 [70]	Decreased productivity due to ineffective communication and increased procrastination (distractions). Increased performance with social support but decreased performance with lower social support. Decreased work-life balance.

Articles with positive impacts on productivity also demonstrated an improvement in work-life balance (n = 1) and organizational commitment (n = 1). However, mixed results were found for other associated measures within the remaining articles. The five articles demonstrating decreases in productivity and performance reported greater work intensification (n = 1), and stress (n = 1), but a mixed impact on job satisfaction (n = 2), work-life balance (n = 2), and distractions (n = 2). Lastly, 38% (n = 5) of the articles reporting both increases and decreases in productivity and performance also showed mixed results for the associated measures: work intensification (n = 2), job satisfaction (n = 2), and both a mediating effect on work-life balance (n = 1), and stress (n = 1). For example, Dixit and colleagues [46] showed that productivity decreased while work intensification increased initially for WFH employees; however, over time, the authors observed greater productivity and lower work intensification among the workers. A description of the specific main outcomes for each study can be found in Table 8.

## 4. Discussion

Substantial interest surrounding the impacts of WFH has grown in recent years in response to the increasing popularity of flexible work arrangements. Previous research has shown the relationship between WFH and several physical and mental health outcomes [23,76–79], however these typically fall under health and safety issues from a business perspective, which often receive little resources and attention compared to organizational and worker performance and productivity. Therefore, aligning WFH with business goals of organizations may help catalyze awareness from decision makers and serve to effectively implement WFH policies that protect worker health and maintain productivity and performance of both the individual and the organization. By undertaking this research, we have identified the gaps in the literature necessary for future research on the topic and established the groundwork necessary the development of evidence-informed recommendations to assist organizations in ensuring their workforce remains healthy and effective in the transition to a future of WFH arrangements. We have also identified how impacts of WFH vary when the situation surrounding the arrangements differ, which has important implications for organizations as they continue to address the sudden changes to workplaces in response to the COVID-19 pandemic.

The results of our work reveal that the current literature focuses more heavily on how WFH impacts productivity than it does on performance; 27 articles included measures of productivity, while 16 articles measured performance. Further, there is greater emphasis on the effects at the personal-level compared to the organizational-level. This disparity is intriguing and highlights the need for future research to consider the impact of WFH on all levels, particularly for the development of organizational recommendations and policies for WFH that enhance their productivity and performance. This need, and the necessity to understand the interplay between levels has also been voiced by Belanger and colleagues [80] who noted the possibility that the impacts of telecommuting at the individual, group, and organizational level may conflict with one another. Interesting, only nine studies included in our review investigated both the personal and organizational levels, and of those, only three included measures of both productivity and performance.

Additionally, the results of our study identified that most studies were survey-based (n = 23), and asked individuals to self-report on their perceptions of specific metrics, compared to qualitative studies which were interview-style (n = 8) asked participants open-ended questions about their perceptions of specific measures associated with productivity and performance. In total, only 6 articles used an experimental study design. Of these articles, more direct measures of productivity and performance were examined such as attrition rate, and number of tasks completed during a set time frame. Across all articles, regardless of study design, uniform metrics were not used (for example, whether a metrics “score” was determined by 3, 5 or 7 questions). Despite the most common metrics not including productivity or performance (job satisfaction (n = 17), and work-life balance (n = 13)), there was large variability in the combination of metrics used across the studies to evaluate the impact of WFH on productivity and performance. This observation aligns with other research that has emphasized a shortage of experimentally based studies with a consense of metric, noting caution should be taken when inferring causality in non-experimental designs [21].

Further, Belanger and colleagues [80] recognized the importance of time as a factor in understanding the true impacts of WFH, suggesting that the compounding effects of telecommuting over time may change as the experiences of the worker also change. Our review supports this need for longitudinal or experimental studies, as only six were experimental in nature, and only one examined how these outcomes change over time. Through a nine-month experimental WFH study, Bloom and colleagues [5] demonstrated a dramatic 13% performance increase and showed that some improvement observed over the first two months of the study was due to addressing IT and logistical challenges.

### 4.1 COVID-19 considerations

The outcomes from the studies included in our analysis revealed varied and sometimes mixed results. Although other effects of telecommuting are consistent across a variety of studies (i.e., job satisfaction, organizational commitment, stress), moderating factors play an essential role and affect the strength of the impact on outcomes. Overall, 59% of all the articles reported that productivity and/or performance increased while in a WFH arrangement, 14% demonstrated declines and 27% reported mixed results (i.e., both effects) depending on moderating factors (i.e., years of tenure, gender, or caring responsibilities). For example, Feng and Savani [48] found that men reported greater productivity and job satisfaction compared to women when working from home during the pandemic.

In addition to the role that moderating factors may play in contributing to the mixed results, we must acknowledge the different situations which influence the adoption of a WFH arrangement for organizations or individuals. Traditionally, organizations offering WFH options do so because they believe in the value of the practice, they have the technology and other supports in place to enable WFH or employees are requesting to undertake WFH. Many telecommuting arrangements are designed to support employee choice with a combination of days in the office and home through employment negotiations. As such, those who want to WFH are more likely to utilize these programs and report the benefits of the arrangement. However, when WFH becomes a mandatory, full-time requirement, additional factors influence the impacts of such arrangements. The sudden shift to entire organizations and teams working remotely in response to the COVID-19 pandemic must be considered.

Our review process returned 13 articles specifically focused on the impacts of WFH on productivity and performance during the COVID-19 pandemic, all of which had employees working remotely on a full-time, mandatory basis. It is worth noting that of the 24 articles published prior to the onset of COVID-19, none focused on full-time, mandatory WFH. All participants either worked remotely part-time or chose to WFH on a full-time basis. Interestingly, when considering the consensus of WFH impact between these two work arrangement scenarios, only COVID-19 articles reported decreases in productivity and performance. Further, only 23% of the articles published during COVID-19 (and therefore focused on mandatory WFH arrangements) revealed overall positive impacts of WFH on productivity and performance whereas the remaining 77% of the articles demonstrated either mixed results or negative effects. Conversely, 79% of the pre-pandemic articles (and therefore focused on non-mandatory WFH arrangements) showed positive effects whereas the remaining 21% revealed mixed results or no effects. Our findings suggest that the positive impacts that WFH can have on productivity and performance are likely related to non-mandatory arrangements, which supports other previous research. A review by Allen and colleagues [21] found moderate levels of telecommuting provides the most value, providing flexibility and minimizing the impediments to co-worker relationships, knowledge exchange and innovation. Future work in this area must consider the contextual factors leading to WFH arrangements in both the design and analysis of the research and acknowledge the workforce may be managing traditional stressors of WFH, but in the case of COVID-19, also with external stressors likely to influence the impacts on workers [81,82].

Specifically related to WFH during COVID-19, recent research has revealed that significant gender differences emerge when considering the advantages and disadvantages of WFH arrangements. In our work, although gender representation within sample sizes was evenly split (51.9% female, 48.1% male), gender analysis was not considered in many articles. In fact, only two studies explicitly examined the impact of gender. Interestingly, Sherman [75] found that mothers reported greater job performance and decreased work-life conflict when having the option to WFH, whereas Feng and Savani [48] indicated that women reported decreased productivity and lower job satisfaction after WFH became mandatory during COVID-19. These results may be attributed to increased home responsibilities during lockdown situations that pose pressures not normally present when WFH. In broader studies of WFH, Oakman and colleagues [23] revealed that women were less likely to experience improved health outcomes from WFH arrangements. As well, the increased work demands often experienced in WFH settings, such as off-hours messaging between team members impact women more negatively than men [76,83].

### 4.2 Limitations and future research

We must acknowledge the limitations of this work. First, for each of the two core concepts of our search (i.e., WFH terms and productivity/performance), we identified a list of key terms to be included in the search. Given the lack of an internationally recognized definition of the term “telework” [84], and the sudden rise in WFH as a result of the COVID-19 pandemic, new terminology may have emerged, such as the term “work from anywhere” and may have been excluded in the original search. Different research groups may identify different search key terms; therefore, there is always a possibility of excluding certain key terms, resulting in the exclusion of some articles. However, we found a considerable body of literature using our list of key terms, which were generated based on research team’s discussion with a librarian; thus, we believe this common limitation would not have affected our overall results. Second, we limited our search to include articles which specifically indicated measures of productivity and/or performance were used. We chose this approach to ensure that we were not interpreting results on behalf of the authors, however it is possible that articles addressing productivity and performance indirectly, through evaluation of metrics associated with productivity and performance were excluded. Lastly, in response to the COVID-19 pandemic, there has been a sharp rise in interest surrounding the impacts of WFH in the academic literature. It is expected that a significant number of WFH-related research studies will be published as more data becomes rapidly available. Therefore, we recognize that the literature surrounding WFH during COVID-19 included in this review may be limited and we encourage future research groups to add to our interpretations.

Overall, the results of our study find that productivity was more actively studied than performance, and nearly all articles asked employees to self-report on their own productivity rather than asking supervisors or managers to evaluate their employees’ productivity or performance, or using a more direct, quantitative measure. Further, personal productivity was more commonly examined than organizational productivity. Many studies did not specifically examine group differences such as sex and gender, caregiving responsibilities, or job type. Therefore, ample opportunity exists for future research to fill these gaps and contribute to the development of more explicit understanding of productivity and performance at the organizational level, understand how WFH impacts performance-based measures, examine and how these impacts differ between groups.

### 4.3 Implications and recommendations

With entire organizations around the world suddenly required to work remotely as a means of mitigating the spread of COVID-19, organizations and policymakers are working to reduce the impact of such drastic changes on the labour markets. Even when working life begins to return to pre-pandemic “normal”, some form of WFH arrangement is likely to stay, and organizations will need to be prepared to accommodate these arrangements and be equipped with the resources necessary to determine how WFH can work for them. This review lays the groundwork necessary to help organizations develop evidence-informed resources and guidelines to ensure they maintain or optimize their productivity and performance.

When developing a WFH program, organizations should keep mind that WFH is not a one-size-fits-all arrangement. The positive and negative implications of WFH programs on productivity and performance can vary significantly depending on the type of arrangement in question (e.g., full-time, part-time, mandatory, or optional) [67]. Evidence supports that the benefits of WFH, such as maximizing employee satisfaction and productivity, are realized when there is a balance between remote work and in-office work [62]. Prior to the COVID-19 pandemic, research on WFH arrangements showed positive impacts on both organizational and personal productivity and performance. However, far less conclusive, and positive impacts are observed when reviewing the impacts of WFH during the COVID-19 pandemic. These results suggest external factors (e.g., supervision of children, impacts of lockdowns) should be considered when evaluating the effectiveness of a WFH program when it is mandatory. Setting clear goals and expectations for managing the workload of WFH is imperative, as overworking (i.e., work intensification) can be an issue [65], particularly in times of public health crises. Our results are in line with Virick and colleagues [62] who suggests that organizations should consider creating several types of WFH programs for employees that meet different needs.

In addition to the external circumstances leading to WFH arrangements, considerations should be made with respect to the type of jobs and tasks to be completed at home, the team dynamic, and what productivity or performance means to the organization. Jobs not requiring regular teamwork, or in-person facetime are well suited for WFH, as are those where quantity and quality of performance are easily quantifiable or where the link between effort and performance are direct [5]. Golden and Gajendran [50] suggested that job performance can be improved among WFH employees who hold complex jobs, for those who require low levels of interdependence, and for those who require less social support. Furthermore, job duties requiring creativity, rather than duties that involve more dull tasks, can also benefit greatly from a WFH arrangement [61,72]. Regardless of the reason for a WFH program, finding a balance between the physical and virtual contact, when permitted, is important [71]. Where this balance lies depends on the organization, the team, or the project and the requirements that need to be met.

Implementing and evaluating a WFH program will differ across organizations based on their own organizational priorities. However, in general, policies and resources should consider what performance and productivity means to the organization, how it is defined and how it will be measured. For example, when only person-level effects are considered, organizations may wrongly conclude that telework is ineffective regarding certain outcomes [71]. Results from our work support this recommendation. When evaluating the effectiveness of WFH, organizations are encouraged to ensure the breadth of their metrics examine a wide range of factors that can impact productivity and performance. Furthermore, it suggested that organizations (managers, decision makers, policy makers etc.) must consider the impact of remote work not only on those making the switch to WFH, but also in relation to those remaining in the office [68].

Lastly, successful WFH programs requires a culture shift in organizations [85]. Training may be required to ensure all employees view WFH as a standard operating procedure through which work is accomplished, while adhering to a formal written telework policy [85–87]. Managers play an important role in facilitating successful WFH. However, for some managers, changes in their mode of communication or expectations may need to be modified. Training for managers may be required to support any changes necessary to facilitate the transition from more traditional assessment of productivity and performance to one that better suits a WFH arrangement. When viewing telework as a work design initiative, it can boost performance, rather than being primarily a work-family benefit [50]. Managers should receive training to provide accurate support to help change traditional thinking and assessment of performance into goal-oriented management and result oriented systems [40].

## 5. Conclusion

The popularity of WFH arrangements is increasing, and it is anticipated that a significant surge in the number of WFH employees will continue beyond the COVID-19 pandemic, signifying the importance of understanding the productivity and performance outcomes associated with WFH. Overall, the findings from this study suggest that WFH can have positive impacts on personal and organizational productivity and performance. Productivity and performance appear to be impacted differently in WFH situations that are mandatory, such as during the COVID-19 pandemic. It is anticipated that results of the review will be used to create guidelines and recommendations to assist organizations with facilitating and evaluating an optimal WFH arrangement for their employees that promotes productivity and performance.

## Supporting information

S1 Checklist PRISMA checklist. (DOC)

S1 Dataset Minimumal dataset. (XLSX)
